# Supplementary material for: Secretory GFP reconstitution labeling of neighboring cells interrogates cell–cell interactions in metastatic niches
Source: Nat Commun. 2023 Dec 5;14:8031. doi: 10.1038/s41467-023-43855-2 (PMC10697979; doi:10.1038/s41467-023-43855-2)
Supplement: Supplementary file 1 — Supplementary Information [file 41467_2023_43855_MOESM1_ESM.pdf]

Supplementary Information

Secretory GFP reconstitution labeling of neighboring cells interrogates cell–cell interactions in metastatic niches

Minegishi et al.

-Supplementary Figures

-Supplementary Tables

-Supplementary Methods

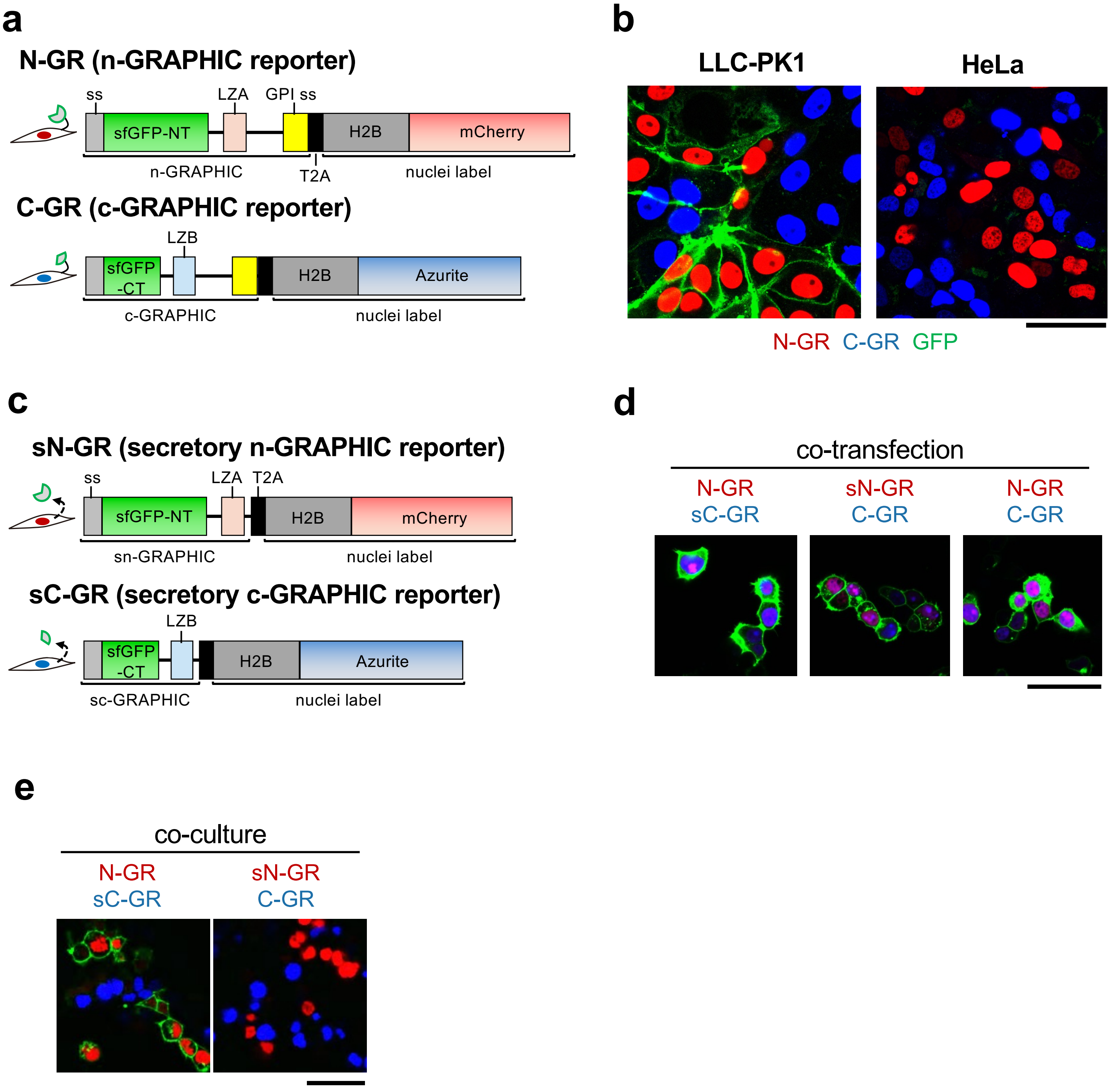

Supplementary Figure 1. **Design and construction of *s*GRAPHIC system. a** GRAPHIC reporter genes. Reporter genes encode molecules consist of signal sequence (ss), N-terminal (NT) or C-terminal (CT) of split superfolder GFP (sfGFP) fragment, leucine zipper domain A (LZA), leucine zipper domain B (LZB), and GPI attachment signal sequence (GPI ss). They also encode H2B-mCherry and H2B-Azurite that are expressed in the nuclei to identify and estimate the expression of N-terminal and C-terminal sfGFP fragments, respectively. **b** GRAPHIC labeling in co-culture of LLC-PK1/N-GR and LLC-PK1/C-GR or HeLa/N-GR and HeLa/C-GR. **c** Design of *s*GRAPHIC and GRAPHIC reporter genes. **d** Co-expression of the indicated *s*GRAPHIC and GRAPHIC reporter genes in HEK293T cells. Green indicates GFP reconstituted on the cell surface. Similar results were observed in multiple fields of view in independent duplicate experiments. A scale bar indicates 50 μm. **e** HEK293T cells expressing sC-GR or C-GR (blue) and HEK293T cells expressing N-GR or sN-GR (red) were co-cultured, and GFP reconstituted on the cell surface is shown in green. Similar results were observed in multiple fields of view in independent duplicate experiments. A scale bar indicates 50 μm.

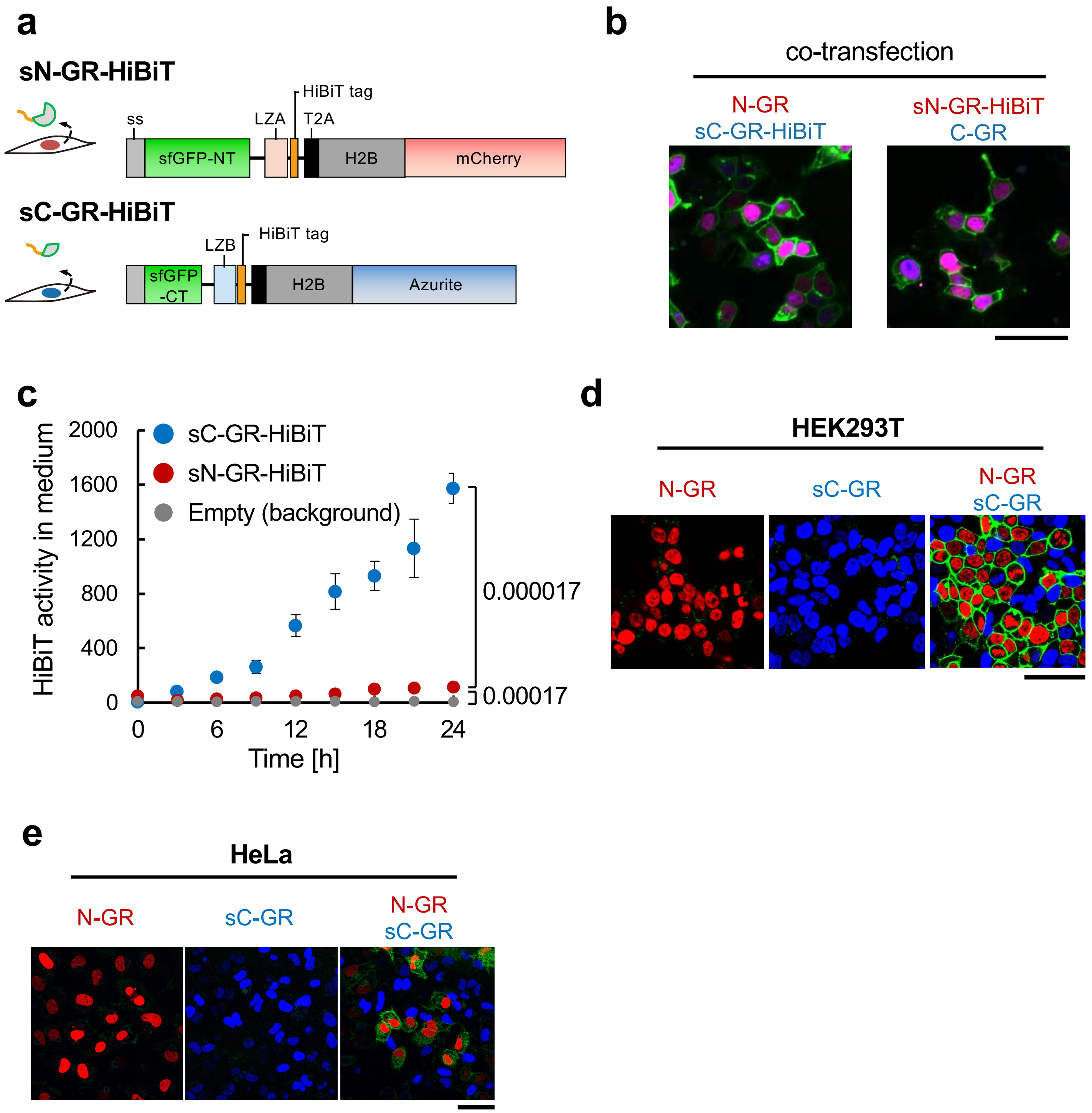

Supplementary Figure 2. **Characterization of *s*GRAPHIC system in cells. a** Reporter design for assessment of GFP fragments secretion. HiBiT tag (VSGWRLFKKIS) was conjugated to C-terminal of secretory GFP fragments. **b** HEK293T cells express indicated HiBiT tagged reporter genes. Green indicates GFP reconstituted on the cell surface. Similar results were observed in multiple fields of view in independent duplicate experiments. A scale bar indicates 50 μm. **c** The HiBiT reporter genes were transfected into HEK293T cells, and the luminescence activity of HiBiT in the culture medium was measured every 3 hours for 24 hours. Data were statically analyzed with Holm-Sidak adjusted multiple t-test (n = 3 biologically independent samples). The p-values are indicated in the graph. Source data are provided as a Source Data file. **d** *s*GRAPHIC labeling in HEK293T cells. *s*GRAPHIC specifically labeled HEK293T/N-GR (red nuclei) with GFP (green) when co-cultured with HEK293T/sC-GR cells (blue nuclei). Similar results were observed in multiple fields of view in independent triplicate experiments. A scale bar indicates 50 μm. **e** *s*GRAPHIC labeling in HeLa cells. *s*GRAPHIC specifically labeled HeLa/N-GR (red nuclei) with GFP (green) when co-cultured with HeLa/sC-GR cells (blue nuclei). Similar results were observed in multiple fields of view in independent duplicate experiments. A scale bar indicates 50 μm.

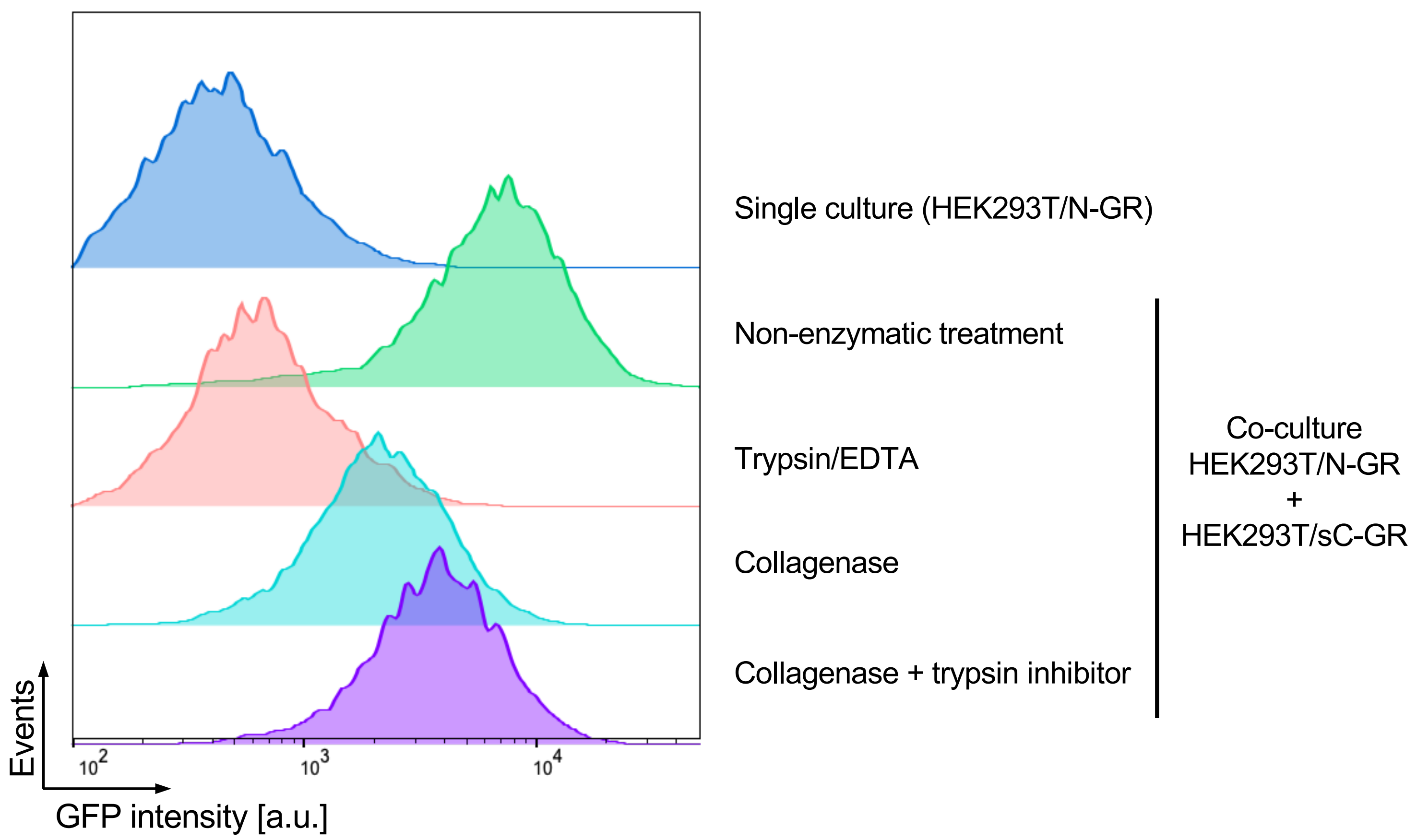

Supplementary Figure 3. **Flow cytometry histogram of GFP fluorescence retained in cells.** Twenty-four hours after co-culturing with HEK293T/sC-GR, HEK293T/N-GR was harvested by treatment with non-enzymatic solution, trypsin/EDTA, collagenase, or collagenase plus trypsin inhibitor. Their GFP fluorescence intensity was measured by flow cytometry. Similar results were observed in independent duplicate experiments.

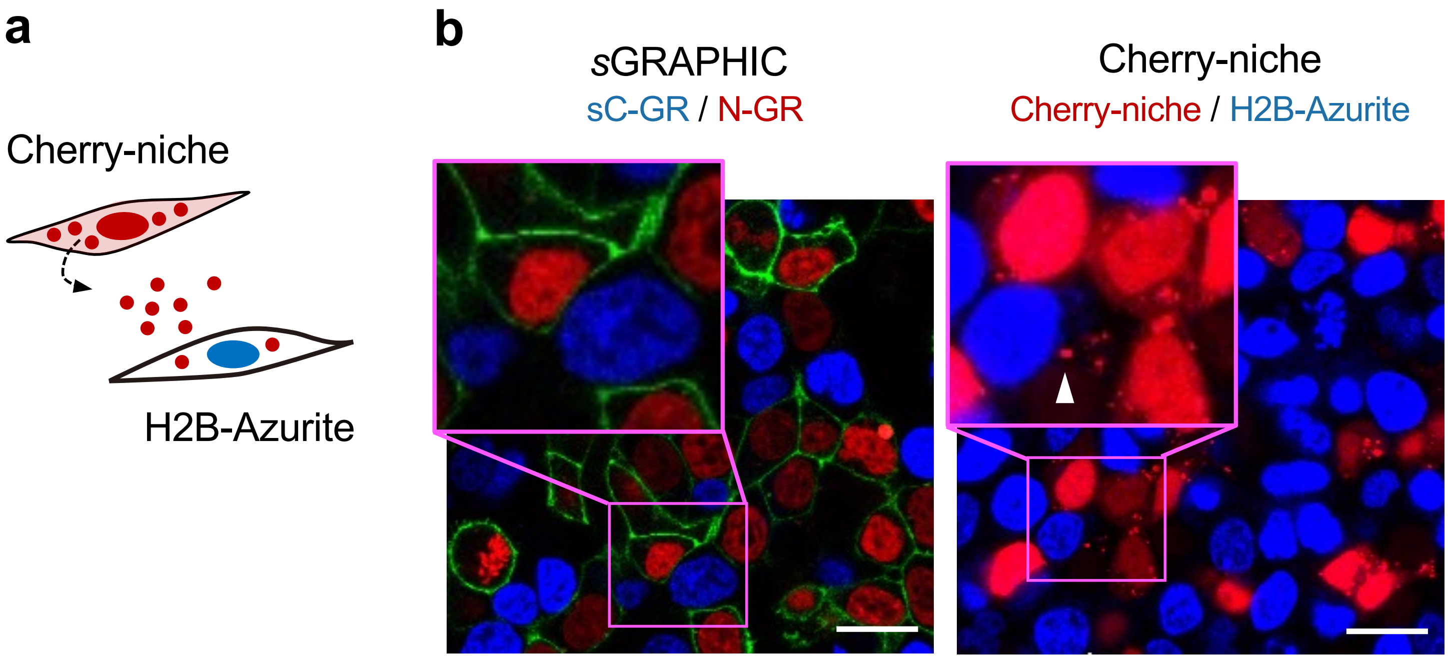

Supplementary Figure 4. **Fluorescence labeling of neighboring cells by *s*GRAPHIC and Cherry-niche. a** Schematic of Cherry-niche labeling by HEK293T cells expressing Cherry-niche or H2B-Azurite. **b** Confocal fluorescent observation of *s*GRAPHIC and Cherry-niche labeling 24 hours after co-culture of HEK293T stable expressing indicated reporter genes. A white arrowhead indicates mCherry punctum transferred into HEK293T/H2B-Azurite cells. Similar results were observed in independent duplicate experiments. Scale bars indicate 20 μm.

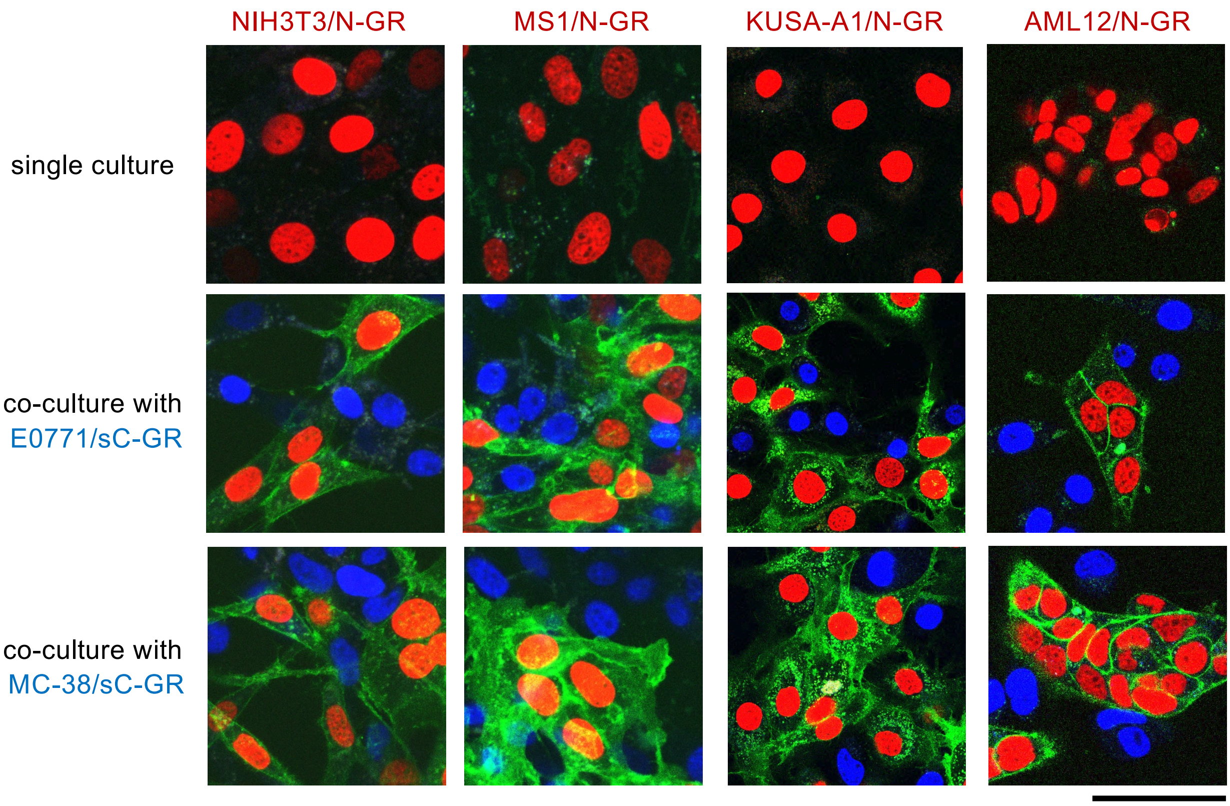

Supplementary Figure 5. **Fluorescent confocal microscope observation of *s*GRAPHIC labeling of cancer cell–tissue-resident cell interactions.** Various tissue-resident cells stably expressing N-GR were co-cultured with E0771/sC-GR or MC-38/sC-GR in the ratio of 1:1 for 24 hours. Similar results were observed in multiple fields of view in independent duplicate experiments. A scale bar indicates 50 µm.

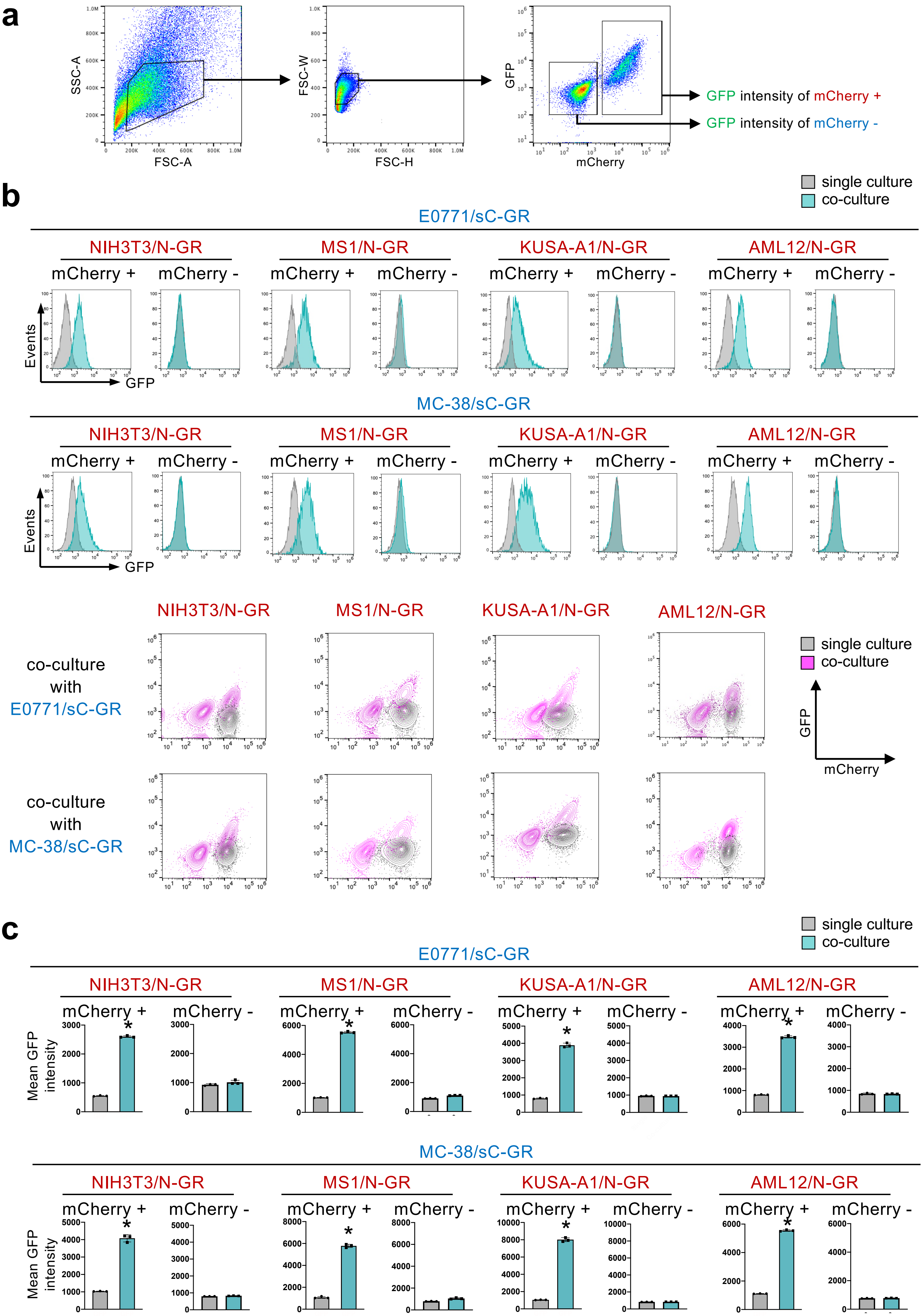
a
SSC-A
FSC-A
FSC-W
FSC-H
GFP
mCherry
GFP intensity of mCherry +
GFP intensity of mCherry -
b
single culture
co-culture
E0771/sC-GR
NIH3T3/N-GR
MS1/N-GR
KUSA-A1/N-GR
AML12/N-GR
mCherry +
mCherry -
Events
GFP
MC-38/sC-GR
co-culture with E0771/sC-GR
co-culture with MC-38/sC-GR
c
Mean GFP intensity

Supplementary Figure 6. **Flow cytometry analysis of *s*GRAPHIC labelling. a** Gating strategy for flow cytometry analysis of *s*GRAPHIC labeled cells. **b** Representative flow cytometry plots of tissue-resident cells in single culture (gray) and co-culture with cancer cells (blue-green / magenta). Similar results were observed in independent duplicate experiments. **c** Quantitative analysis of mean GFP intensity of single culture (gray) and co-culture (blue-green) in flow cytometry analysis of b. Data were statistically analyzed with two-tailed Student's t-test (n = 3 biologically independent samples, *p<0.0001). Source data are provided as a Source Data file.

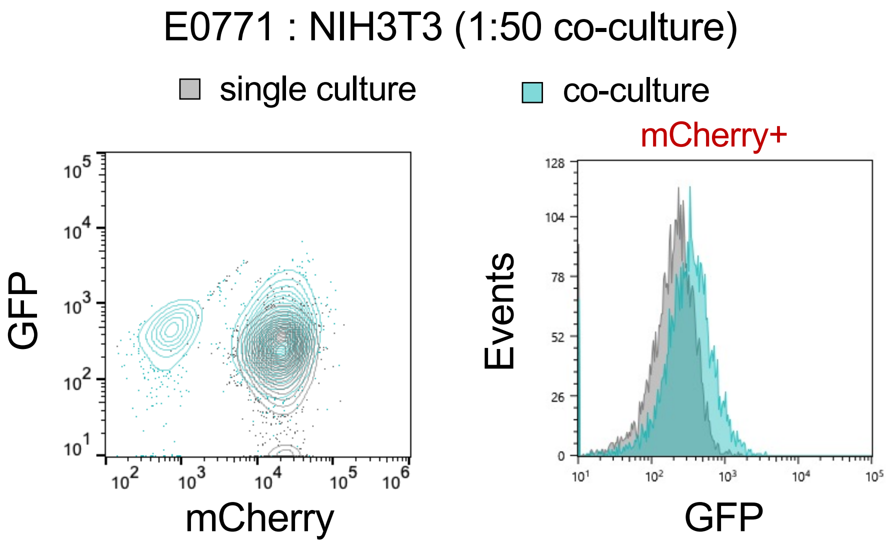

Supplementary Figure 7. **Flow cytometry analysis of *s*GRAPHIC labeling by a small number of cancer cells.** We co-cultured NIH3T3/N-GR and E0771/sC-GR at a ratio of 50:1**.** A representative plot and GFP histogram from NIH3T3 single culture (gray) and co-culture (green) samples are shown. Similar results were observed in independent duplicate experiments.

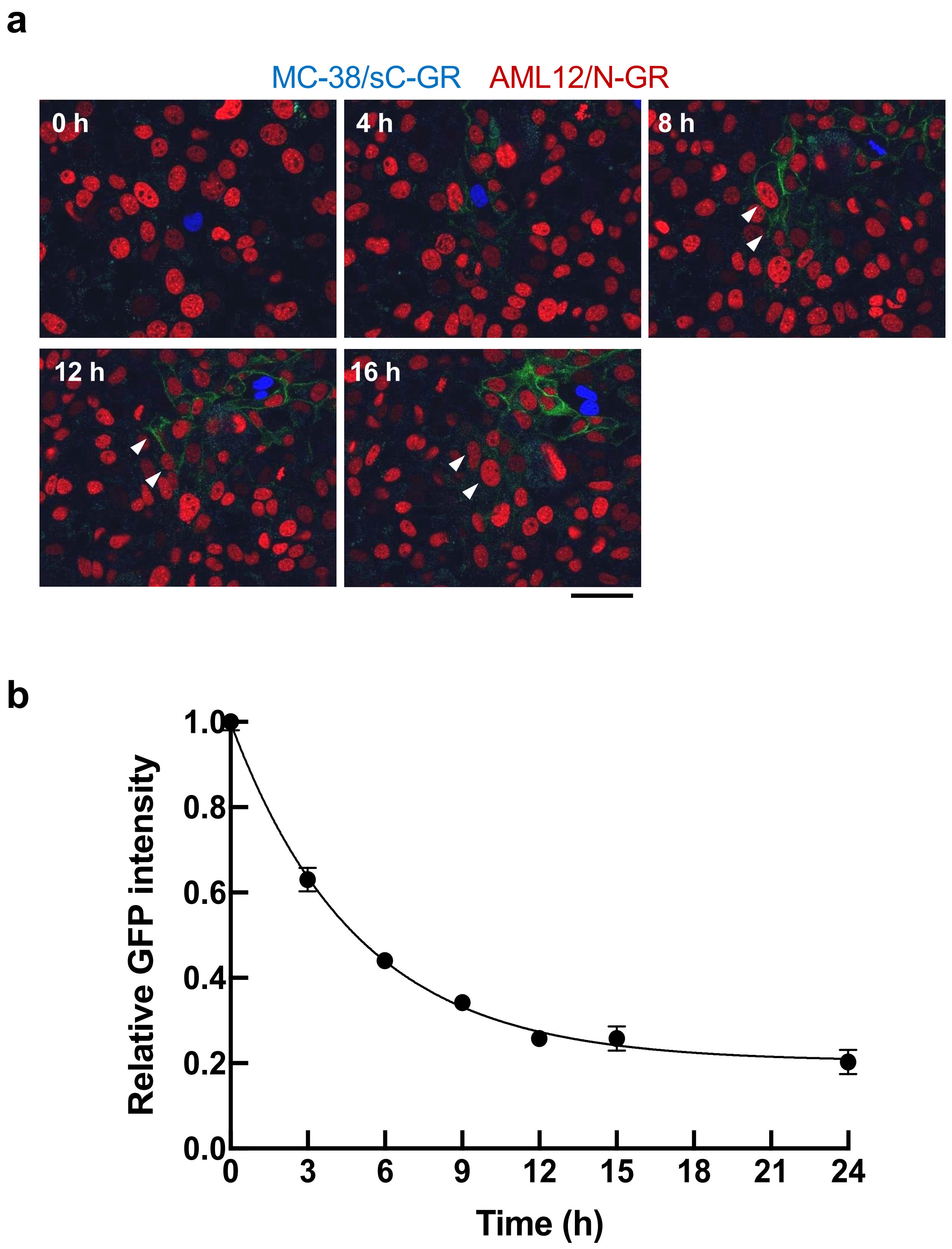

Supplementary Figure 8. **Time-course characterization of *s*GRAPHIC labeling. a** Snapshots of time-lapse fluorescence imaging in co-culturing MC-38/sC-GR and AML12/N-GR at 1:50 from Supplementary Video 3. White arrow heads indicate cells labeled with GFP at 8 h. Similar results were observed in independent duplicate experiments. A scale bar indicates 50 μm. **b** The half-life of reconstituted GFP on NIH3T3/N-GR after co-culturing with E0771/sC-GR. The GFP intensity of GFP-labeled NIH3T3/N-GR was measured over time by flow cytometry and the relative intensities are shown (n = 3 biologically independent samples for each time point). Source data are provided as a Source Data file.

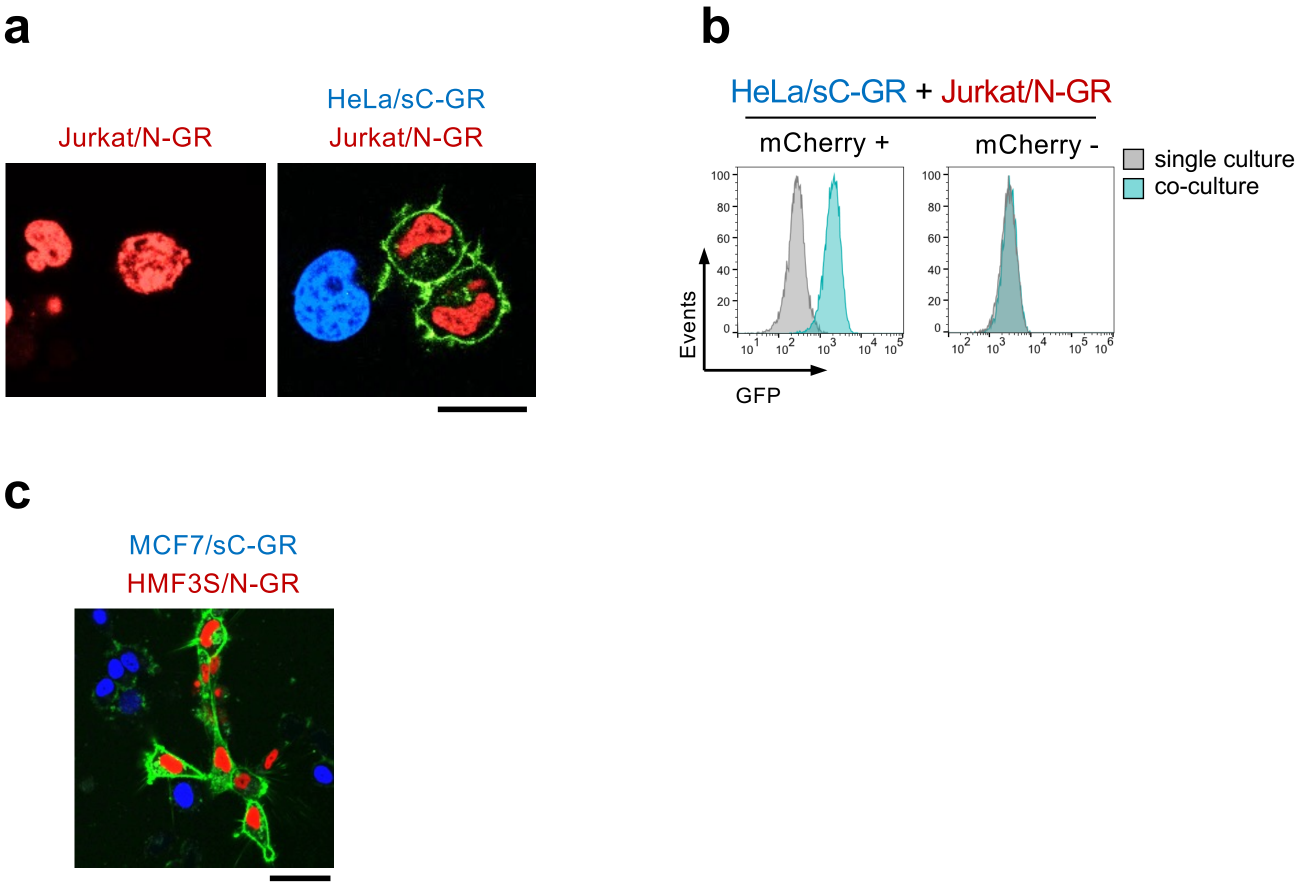

Supplementary Figure 9. ***s*GRAPHIC labeling of cell-cell interactions in human cell lines.** **a** Fluorescent confocal microscope observations of *s*GRAPHIC labeling of Jurkat/N-GR co-cultured with HeLa/sC-GR. Similar results were observed in independent duplicate experiments. A scale bar indicates 10 μm. **b** Flow cytometry analysis of *s*GRAPHIC labeling of Jurkat/N-GR co-cultured with HeLa/sC-GR at a ratio of 1:1 for 24 hours. Similar results were observed in independent duplicate experiments. **c** A representative fluorescent confocal microscopic image of *s*GRAPHIC labeling of HMF3S/N-GR co-cultured with MCF7/sC-GR. Similar results were observed in independent duplicate experiments. A scale bar indicates 50 μm.

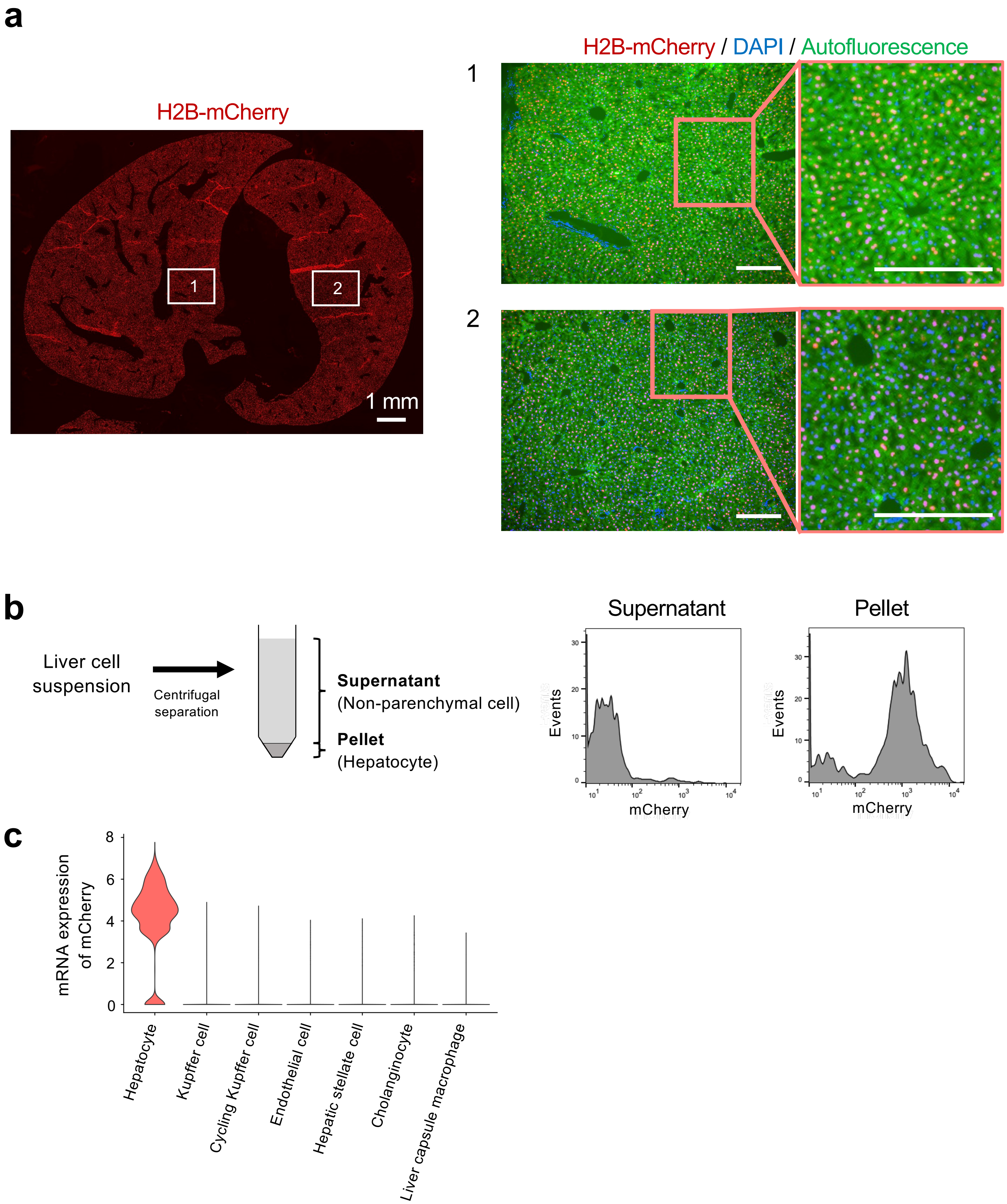

Supplementary Figure 10. **AAV-mediated gene transduction of the *s*GRAPHIC reporter in murine liver tissues. a** Fluorescence microscopy images of H2B-mCherry expression in a tissue section of the murine liver infected with AAV8/N-GR. A scale bar is 1 mm (left panel). High magnification images of areas 1 or 2 in the left panel are shown. Scale bars are 200 μm (right panels). **b** Representative flow cytometry plots for mCherry intensity of liver-resident cells in the supernatant and pellet fraction after centrifugation of liver cell suspension prepared from the AAV8/N-GR-infected mice. **c** scRNA-seq analysis of mCherry expression in the liver-resident cells (hepatocyte, n = 1439; kupffer cell, n = 5743; cycling kupffer cell, n = 1367; endothelial cell, n = 297; hepatic stellate cell, n = 257; cholaginocyte, n = 192; liver capsule macrophage, n = 522).

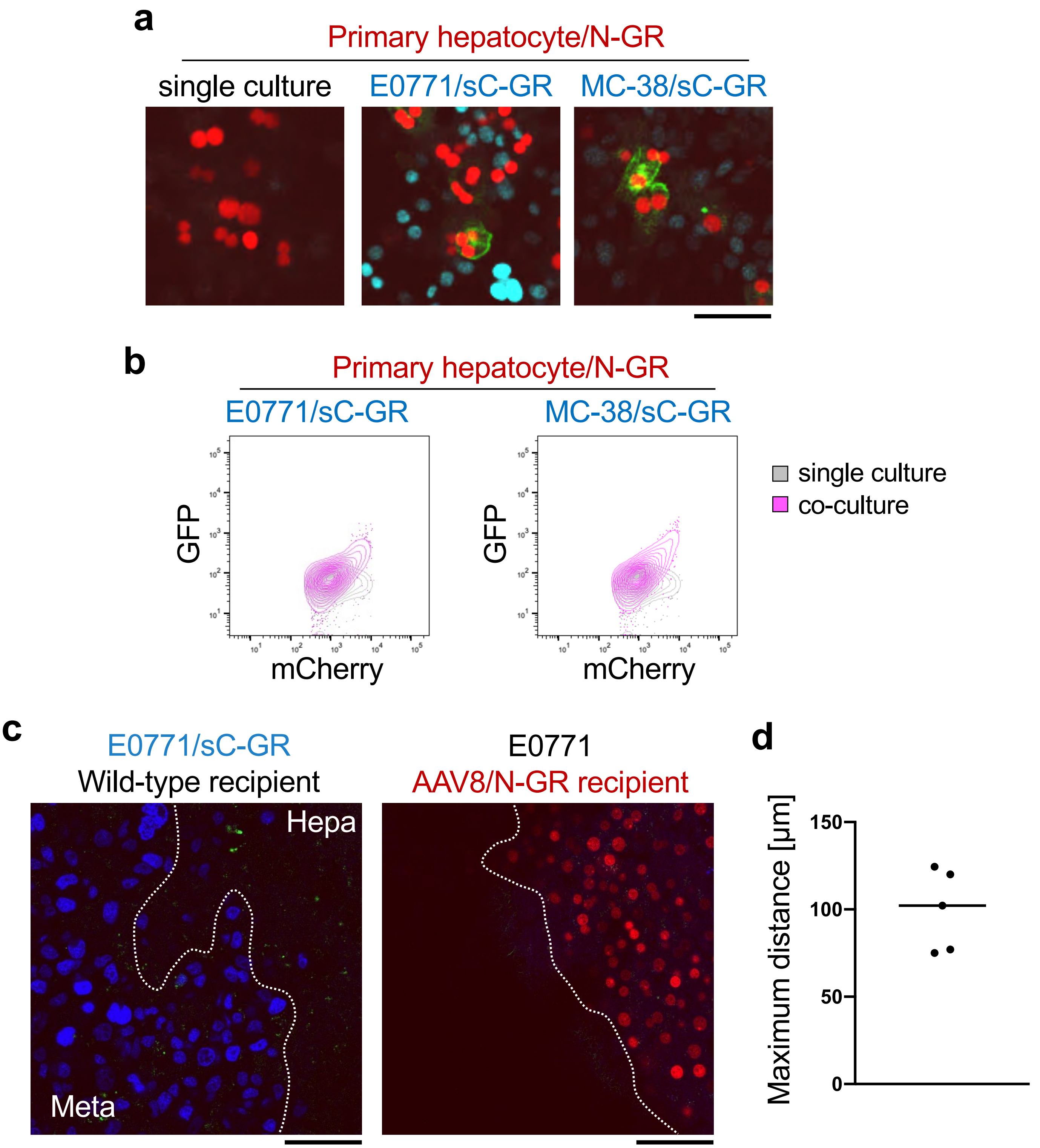

Supplementary Figure 11. **Characterization of *s*GRAPHIC labeling in the liver. a** Confocal microscope observation of *s*GRAPHIC labeling of primary hepatocytes co-cultured with cancer cells. Hepatocytes isolated from the liver of AAV8/N-GR-infected mice were co-cultured with E0771/sC-GR or MC-38/sC-GR. A scale bar indicates 50 μm. **b** Flow cytometry analysis of *s*GRAPHIC labeling of hepatocytes co-cultured with cancer cells. Hepatocytes isolated from the liver of AAV8/N-GR-infected mice were co-cultured with E0771/sC-GR or MC-38/sC-GR. Representative plots of hepatocytes in single culture (gray) and co-culture (magenta) are shown. Similar results were observed in independent duplicate experiments. **c** Confocal fluorescence images of border area of metastatic colony (Meta)-hepatic tissue (Hepa). The metastasized liver tissues were observed at 3 weeks after injecting E0771/sC-GR to wild-type mice (left) or injecting E0771 to mice administered by AAV8/N-GR (right). Scale bars indicate 50 μm. Similar results were observed in independent duplicate experiments. **d** Maximum distance of *s*GRAPHIC labeling in liver metastasis colonies. n = 5.

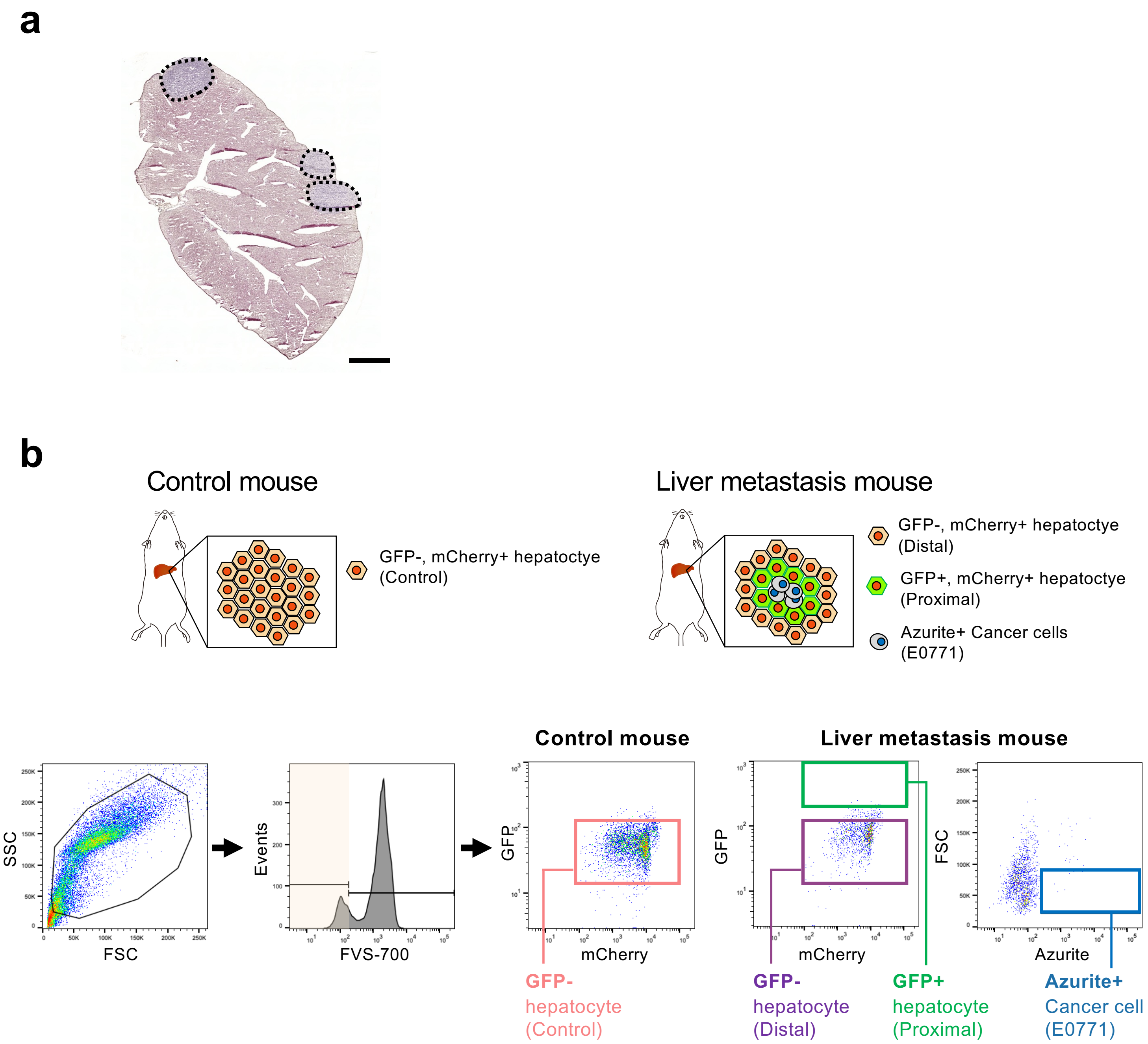

Supplementary Figure 12. **Isolation of metastasis-associated hepatocytes from the murine model of liver metastasis. a** Representative hematoxylin-eosin stained liver section with metastatic colonies three weeks after E0771/sC-GR. Dashed-line areas indicate metastatic colonies. A scale bar indicates 2 mm. **b** Gating strategy for flow sorting of cells from the dissociated healthy and metastasized livers. GFP+ hepatocytes (proximal) and GFP- hepatocyte (distal) from E0771/sC-GR metastasized liver and GFP- hepatocyte (control) from control healthy liver were isolated.

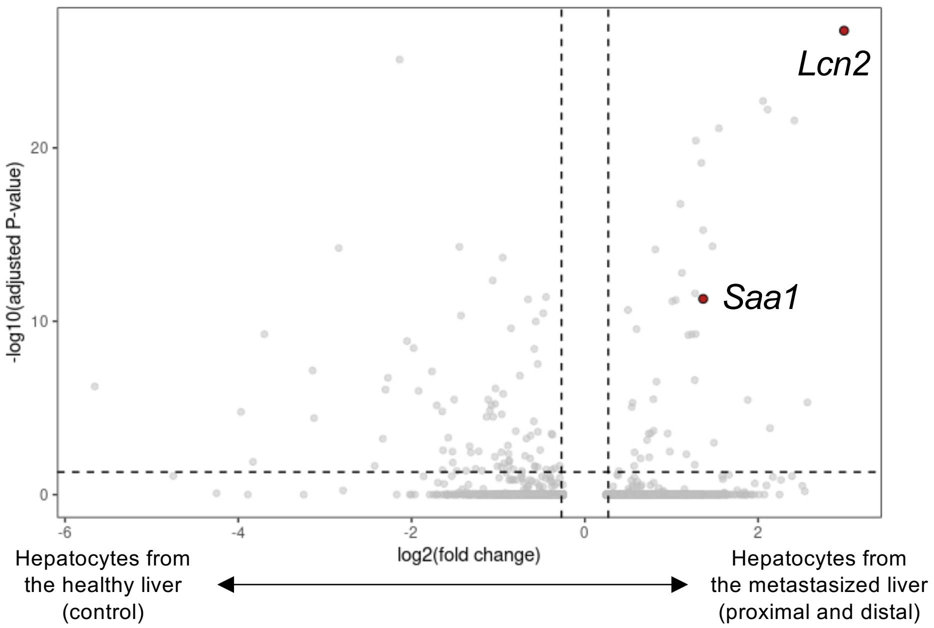

Supplementary Figure 13. **Scatter plot of differential gene expression in hepatocytes harvested from the liver tissues.** Gene expression data were obtained by scRNA-seq of hepatocytes from the metastasized (proximal and distal, n = 158) and those from the healthy livers (control, n = 87). The vertical dot lines correspond to a log 2-fold change of -0.27 and 0.27, while the horizontal dot line indicates an adjusted P-value of 0.5. (two-sided version of Wilcoxon Rank-Sum test).

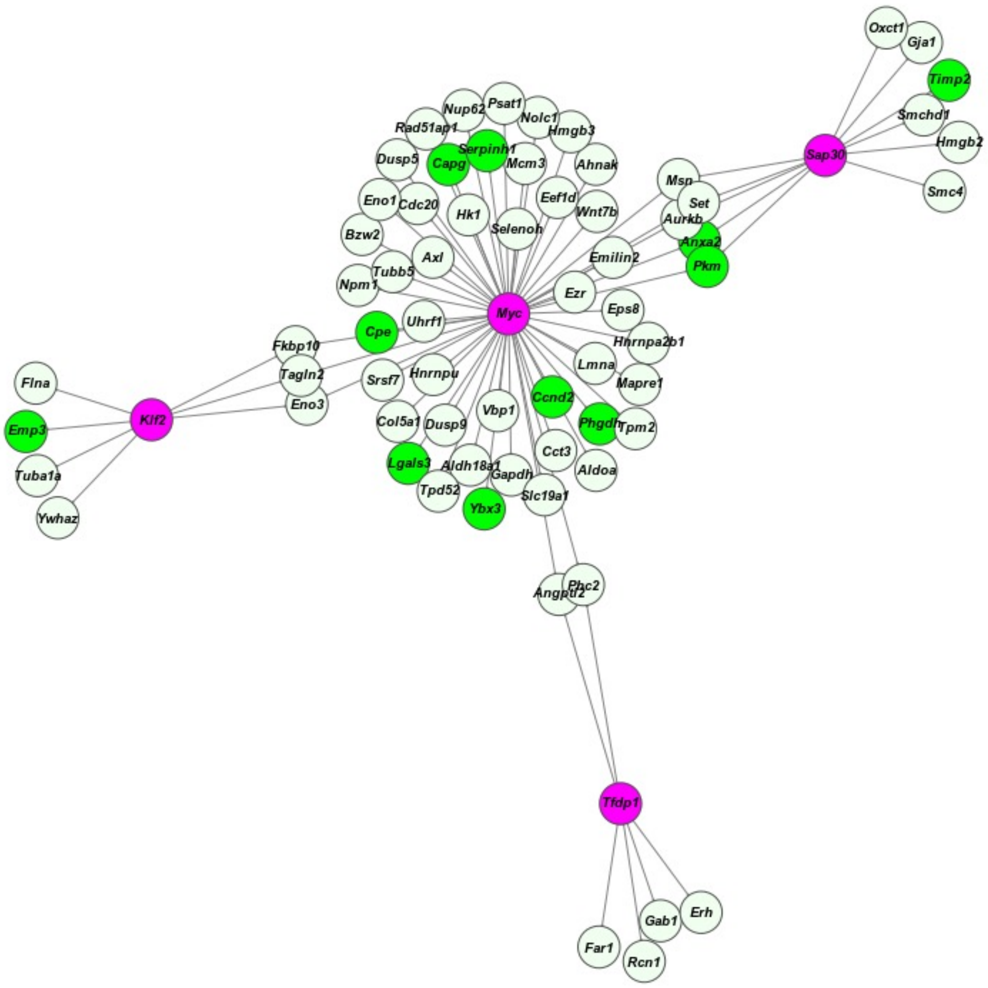

Supplementary Figure 14. **Gene regulatory network (GRN) in metastasis-associated hepatocytes.** GRNs associated with *Myc* in the cluster 3 hepatocytes in Figure 4a were inferred by single-cell regulatory network inference and clustering (SCENIC, 1.2.4[68,69]) workflow. The resulted GRN in the cluster 3 was visualized with marker genes (418 genes) satisfying adjusted p-value < 0.01 (two-sided version of Wilcoxon Rank-Sum test), average log 2-fold change > 1, CoExWeight > 0.007 with *Myc*, *Klf2*, *Tfdp1*, *Sap30*, *E2f4* using CRAN igraph (1.3.2). The transcription factors and marker genes of the cluster 3 listed in Figure 4b were highlighted with magenta and neon green, respectively.

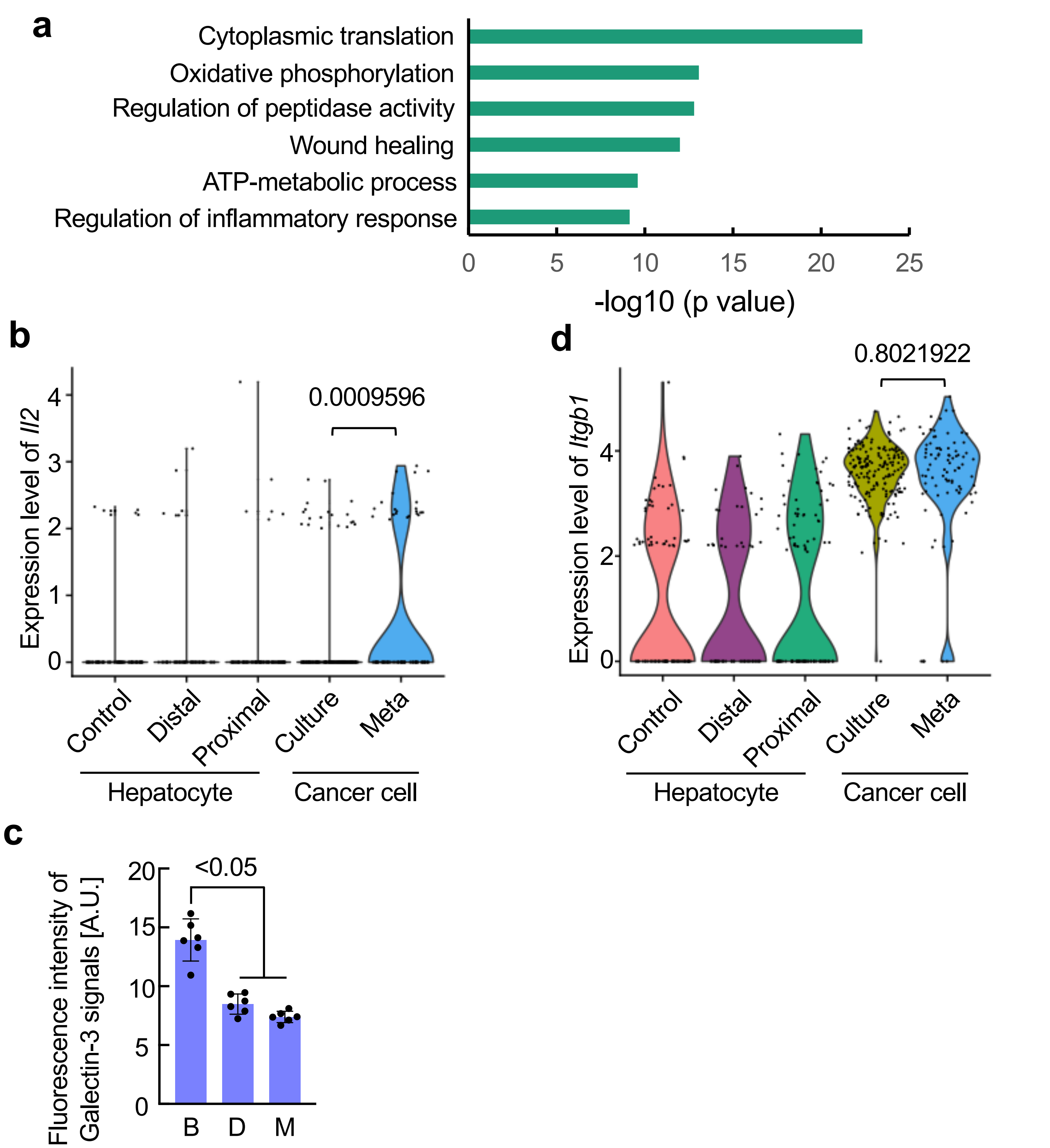

Supplementary Figure 15. **Gene regulation in cancer cells and hepatocytes in liver metastasis. a** Gene ontology of up-regulated genes (adjusted P-value < 0.1, log 2-fold change absolute value > 0.3) in E0771 from the metastasized liver as compared to from a culture dish. **b** Expression levels of interleukin 2 (*Il2*) in hepatocytes (control, n = 87; distal, n = 59; proximal, n = 99) and E0771 cancer cells (culture, n = 189; distal, n = 59; proximal, n = 99) by scRNA-seq. Data were statistically analyzed with analysis of variance and Tukey honestly significant difference test (95% family-wise confidence level). The adjusted p-value is shown in the graph. **c** Galectin-3 signals in immunofluorescent staining images of tissue–metastasized colonies border (B), distal liver tissue (D) and non-peripheral area of the metastatic colony (M). Data were statically analyzed with multiple test (n=6). The p-value is shown in the graph. Source data are provided as a Source Data file. **d** Expression levels of integrin β1 (*Itgb1*) in hepatocytes (control, n = 87; distal, n = 59; proximal, n = 99) and E0771 cancer cells (culture, n = 189; distal, n = 59; proximal, n = 99) by scRNA-seq. Source data are provided as a Source Data file. Data were statistically analyzed with analysis of variance and Tukey honestly significant difference test (95% family-wise confidence level). The adjusted p-value is shown in the graph.

Supplementary Table 1. **Cell lines stably transduced with *s*GRAPHIC reporters in this study.**

Reporter	Cell Line	Type	Species
N-GR	HEK293T	Epithelial	Human
	NIH3T3	Fibroblast	Mouse
	MS1	Endothelial	Mouse
	KUSA-A1	Osteoblast	Mouse
	AML12	Hepatocyte	Mouse
	Jurkat	Acute T cell leukemia	Human
	HMF3S	Mammary fibroblast	Human
sC-GR	HEK293T	Epithelial	Human
	HeLa	Cervix adenocarcinoma	Human
	E0771	Breast carcinoma	Mouse
	MC-38	Colon carcinoma	Mouse
	MCF7	Breast carcinoma	Human

Supplementary Table 2. **Reagents for liver dissociation in primary hepatocytes isolation.**

**Solution**	**Reagent**	**Volume**
Perfusion buffer	NaCl (FUJIFILM Wako Pure Chemical Corporation)	8 g
	KCl (FUJIFILM Wako Pure Chemical Corporation)	0.4 g
	$NaH_2PO_4 \cdot 2H_2O$ (FUJIFILM Wako Pure Chemical Corporation)	78 mg
	$Na_2HPO_4 \cdot 12H_2O$ (FUJIFILM Wako Pure Chemical Corporation)	302 mg
	HEPES (Sigma-Aldrich)	2.38 g
	EGTA (Nacalai Tesque)	0.19 g
	$NaHCO_3$ (FUJIFILM Wako Pure Chemical Corporation)	0.35 g
	D-Glucose (FUJIFILM Wako Pure Chemical Corporation)	0.9 g
Total	MiliQ	1000 mL, pH7.2
Hanks Solution	Hanks (Nissui)	9.8 g
	HEPES (Sigma-Aldrich)	2.38 g
	$CaCl_2 \cdot 2H_2O$ (FUJIFILM Wako Pure Chemical Corporation)	0.54 g
	$NaHCO_3$ (FUJIFILM Wako Pure Chemical Corporation)	0.35 g
Total	MiliQ	1000 mL
Digestion buffer	Collagenase (FUJIFILM Wako Pure Chemical Corporation)	25 mg
	Trypsin inhibitor (Nacalai Tesque)	5 mg
Total	Hanks Solution	50 mL, pH 7.5

Supplementary Methods

Abbreviation list

AAV8: Adeno-associated virus serotype 8
ATCC: American Type Culture Collection
DEGs: Differentially expressed genes
DMEM: Dulbecco's Modified Eagle's medium
FBS: Fetal bovine serum
FC: Fold change
Gapdh: Glyceraldehyde-3-phosphate dehydrogenase
GFP: Green fluorescent protein
GPI: Glycosylphosphatidylinositol
GRAPHIC: Glycosylphosphatidylinositol anchored reconstitution-activated proteins to highlight intercellular connections
GRASP: GFP reconstitution across synaptic partners
HUNTER-seq: Highlighting unknown neighbors through extracellular-gfp reconstitution and sequencing
MAHs: Metastatic niche-associated hepatocyte
PBS: Phosphate-buffered saline
PBS-T: PBS containing 0.05% Tween-20
PCA: Principal component analysis
PEI: Polyethyleneimine
RPMI: Roswell Park Memorial Institute-1640
scRNA-seq: Single-cell RNA sequencing
SEM: Standard error of the mean
***s*GRAPHIC**: Secretory GRAPHIC
t-SNE: t-Distributed stochastic neighbor embedding
UMI: Unique molecular identifier

Transient GFP reconstitution assay.

*s*GRAPHIC or GRAPHIC reporters were transduced into HEK293T cells (CloneTech, Mountain View, CA, USA) using Polyethyleneimine (PEI) MAX (Polysciences, Warrington, PA, USA). After 24 hours of culture, the cells were observed with a confocal microscope Zeiss LSM 780 (Carl Zeiss, Oberkochen, Germany).

HiBiT assay for GFP fragment secretion.

*s*GRAPHIC-HiBiT reporter CSII-CMV vectors were transfected into HEK293T cells (CloneTech) by PEI MAX (Polysciences). After overnight culture, the culture medium was replaced with a fresh one, and medium supernatant was harvested every 3 hours for 24 hours. HiBiT activity in the supernatant was measured after 15 minutes incubation of 10 µL the supernatant and 10 µL Nano- Glo® HiBiT Extracellular Reagent (Promega, Madison, WI, USA) using a luminometer GL-210 (MICROTEC, Chiba, Japan).

Half-life measurement of reconstituted GFP by *s*GRAPHIC labeling.

NIH3T3/N-GR cells and E0771/sC-GR cells (3.0×10^6 cells each) were co-cultured for 24 hours and then were harvested with Cell Dissociation Solution (Biological Industries, Cromwell, CT, USA). GFP-positive NIH3T3/N-GR cells were sorted by flow

sorter SH800 (SONY, Tokyo, Japan) and re-seeded on a 24 well-plate. Time-course changes of GFP intensity were measured by flow cytometry SH800 (SONY).

Isolation of non-parenchymal cells from the murine liver.

The supernatant obtained after centrifugation of hepatocytes was harvested and was further centrifuged at 500 g for 10 minutes at 4ºC. After centrifugation, cells were suspended in 1 mL of sterile water for hemolysis and then 1 mL of 2× PBS was added. Non-parenchymal cells were isolated by centrifugation at 400 g for 7 minutes at 4ºC.

Analysis of gene transduction into liver cells.

To visualize N-GR expression, the frozen liver sections were fluorescently observed with fluorescent microscope BZ-X700 (Keyence, Osaka, Japan). The primary hepatocytes and non-parenchymal cells were each harvested from the liver transduced with N-GR, and the mCherry intensity of the harvested cells was analyzed with FACSAria ™ (Becton, Dickinson and Company, Sparks, MD, USA). After dead cells were excluded with signals of Fixable Viability Stain 700 (BD Biosciences, San Jose, CA, USA), major populations of hepatocytes (mCherry-positive) or non-parenchymal cells (mCherry-negative) were respectively gated to harvest them in cell sorting. The isolated hepatocytes were washed twice with PBS containing 0.04% bovine serum albumin

(Thermo Fisher Scientific, Waltham, MA, USA) by centrifugation at 80 g for 5 minutes at 4ºC. The isolated non-parenchymal cells were washed twice with PBS containing 0.04% bovine serum albumin (Thermo Fisher Scientific) by centrifugation at 500 g for 5 minutes at 4ºC. The libraries of hepatocytes and non-parenchymal cells were yielded separately using Chromium Next GEN Single Cell 3' v3.1 and Chromium Controller (10x Genomics, Pleasanton, CA, USA), and sequenced with 150 bp pair-end read using HiSeqX platform (Illumina, San Diego, CA, USA). The reference data for the analysis was created with the *mkref* function of Cell Ranger (6.1.0) (10x Genomics) with a reference genome (mus musculus (GRCm38) and N-GR) and Mus_musculus.GRCm38.102.gtf integrated with N-GR. The fastq files were processed by Cell Ranger (6.1.0) (10x Genomics) with the reference data. Cells with less than 1000 UMI counts were discarded. scRNA-seq data were analyzed using the Seurat R package (4.1.0) [1]. We filtered out cells with more than 10% mitochondrial gene expression or more than 30,000, less than 1000 unique transcripts from the analysis. The counts were normalized with a log-normal transformation and scaled with a scale factor of 100,000. The principal component analysis (PCA) on the single-cell expression matrix was performed with the *RunPCA* function in Seurat with variable genes that were identified with the *VariableFeatures* function *in* Seurat. On the basis of known cell-type markers [2,]

[3, 4, 5, 6], hepatocytes, endothelial cells, Kupffer cells, cycling Kupffer cells, hepatic stellate cells, cholangiocytes, liver capsule macrophages, and T cells were identified. The *VlnPlot* function in Seurat was used for visualizing expression levels of N-GR.

Distance measurement of *s*GRAPHIC labeling.

We measured the distance between the nuclei of the furthest GFP-positive hepatocytes and the nuclei of the most marginal cancer cells in confocal fluorescence images with relatively well-defined boundaries in the metastatic colony–hepatic tissue. The fluorescence images were analyzed with NIH ImageJ/Fiji open-source software.

Supplementary References

1. Hao Y*, et al.* Integrated analysis of multimodal single-cell data. *Cell* **184**, 3573-3587 e3529 (2021).

2. Halpern KB*, et al.* Paired-cell sequencing enables spatial gene expression mapping of liver endothelial cells. *Nat Biotechnol* **36**, 962-970 (2018).

3. Wang Z-Y*, et al.* Single-cell and bulk transcriptomics of the liver reveals potential targets of NASH with fibrosis. *Sci Rep* **11**, 19396 (2021).

4. Macparland SA*, et al.* Single cell RNA sequencing of human liver reveals distinct intrahepatic macrophage populations. *Nature Communications* **9**, 4383 (2018).

5. Aizarani N*, et al.* A human liver cell atlas reveals heterogeneity and epithelial progenitors. *Nature* **572**, 199-204 (2019).

6. Franzén O, Gan L-M, Björkegren JLM. PanglaoDB: a web server for exploration of mouse and human single-cell RNA sequencing data. *Database* **2019**, baz046 (2019).
