# Supplementary material for: Secretory GFP reconstitution labeling of neighboring cells interrogates cell–cell interactions in metastatic niches
Source: Nat Commun. 2023 Dec 5;14:8031. doi: 10.1038/s41467-023-43855-2 (PMC10697979; doi:10.1038/s41467-023-43855-2)
Supplement: Supplementary file 3 — Reporting Summary [file 41467_2023_43855_MOESM3_ESM.pdf]

nature portfolio

Corresponding author(s): Takahiro Kuchimaru, Hirofumi Shintaku

Last updated by author(s): Oct 17, 2023

Reporting Summary

Nature Portfolio wishes to improve the reproducibility of the work that we publish. This form provides structure for consistency and transparency in reporting. For further information on Nature Portfolio policies, see our Editorial Policies and the Editorial Policy Checklist.

Statistics

For all statistical analyses, confirm that the following items are present in the figure legend, table legend, main text, or Methods section.

n/a	Confirmed	
☐	☒	The exact sample size (*n*) for each experimental group/condition, given as a discrete number and unit of measurement
☐	☒	A statement on whether measurements were taken from distinct samples or whether the same sample was measured repeatedly
☐	☒	The statistical test(s) used AND whether they are one- or two-sided<br>*Only common tests should be described solely by name; describe more complex techniques in the Methods section.*
☒	☐	A description of all covariates tested
☐	☒	A description of any assumptions or corrections, such as tests of normality and adjustment for multiple comparisons
☐	☒	A full description of the statistical parameters including central tendency (e.g. means) or other basic estimates (e.g. regression coefficient) AND variation (e.g. standard deviation) or associated estimates of uncertainty (e.g. confidence intervals)
☐	☒	For null hypothesis testing, the test statistic (e.g. *F*, *t*, *r*) with confidence intervals, effect sizes, degrees of freedom and *P* value noted<br>*Give P values as exact values whenever suitable.*
☒	☐	For Bayesian analysis, information on the choice of priors and Markov chain Monte Carlo settings
☒	☐	For hierarchical and complex designs, identification of the appropriate level for tests and full reporting of outcomes
☒	☐	Estimates of effect sizes (e.g. Cohen's *d*, Pearson's *r*), indicating how they were calculated

Our web collection on statistics for biologists contains articles on many of the points above.

Software and code

Policy information about availability of computer code

Data collection	Microscope data were acquired by LAS X 2.0.1(Leica SP8), ZEN2 (Ziess LSM 780), FV31S-SW2.6(Olympus FV3000) and BZ-H3A (Keyence BZ-X700). Flow cytometry data were acquired by Cell Sorter Software (SONY SH800) and BD FACSDiva8.0.2 (Becton, Dickinson and Company FACS Aria)
Data analysis	Plotting data and statistical analyses were performed with GraphPad PRISM 8. Microscopy images were analyzed with ImageJ2 2.3.0. Flow cytometry data were analyzed with FlowJo 10.8.1. Single-cell RNA sequencing data were analyzed with Seurat R package 4.1.0. All code used for analyzing the data is described in the Methods section.<br>Custom code for HUNTER-seq analysis is available on GitHub: https://github.com/Minegishi-Misa/sGRAPHIC-HUNTER.git.

For manuscripts utilizing custom algorithms or software that are central to the research but not yet described in published literature, software must be made available to editors and reviewers. We strongly encourage code deposition in a community repository (e.g. GitHub). See the Nature Portfolio guidelines for submitting code & software for further information.

Data

Policy information about availability of data

All manuscripts must include a data availability statement. This statement should provide the following information, where applicable:

- Accession codes, unique identifiers, or web links for publicly available datasets
- A description of any restrictions on data availability
- For clinical datasets or third party data, please ensure that the statement adheres to our policy

All raw single-cell RNA sequencing data are available in Sequencing Read Archive under accession PRJNA841462. The plasmids will be made available through Addgene upon publication.

Research involving human participants, their data, or biological material

Policy information about studies with human participants or human data. See also policy information about sex, gender (identity/presentation), and sexual orientation and race, ethnicity and racism.

Reporting on sex and gender	n/a
Reporting on race, ethnicity, or other socially relevant groupings	n/a
Population characteristics	n/a
Recruitment	n/a
Ethics oversight	n/a

Note that full information on the approval of the study protocol must also be provided in the manuscript.

Field-specific reporting

Please select the one below that is the best fit for your research. If you are not sure, read the appropriate sections before making your selection.

☒ Life sciences ☐ Behavioural & social sciences ☐ Ecological, evolutionary & environmental sciences

For a reference copy of the document with all sections, see nature.com/documents/nr-reporting-summary-flat.pdf

Life sciences study design

All studies must disclose on these points even when the disclosure is negative.

Sample size	This sample size was chosen based on preliminary experiments indicating that it would be sufficient to detect significant differences in mean.
Data exclusions	No samples were excluded. Genes and cell filtering in scRNA-seq were performed as explained in the method section.
Replication	Number of replicates stated in the figure legends where applicable.
Randomization	Wild-type mice were randomly selected for the experiments. The cell lines were randomly seeded for in vitro assays.
Blinding	No blinding of the experiments was applied because of the nature of experiments. This study did not compare different genotypes and treatments in animal studies. All analysis of histological and cellular experiments were performed automatically by softwares that are not biased toward any samples.

Reporting for specific materials, systems and methods

We require information from authors about some types of materials, experimental systems and methods used in many studies. Here, indicate whether each material, system or method listed is relevant to your study. If you are not sure if a list item applies to your research, read the appropriate section before selecting a response.

Materials & experimental systems

n/a	Involved in the study	
☐	☒	Antibodies
☐	☒	Eukaryotic cell lines
☒	☐	Palaeontology and archaeology
☐	☒	Animals and other organisms
☒	☐	Clinical data
☒	☐	Dual use research of concern
☒	☐	Plants

Methods

n/a	Involved in the study	
☒	☐	ChIP-seq
☐	☒	Flow cytometry
☒	☐	MRI-based neuroimaging

Antibodies

Antibodies used

Galectin-3 primary antibody (CL8942AP, Clone M3/38, Cedarlane Laboratories)
Goat anti-Rat IgG secondary antibody (A-21094, Invitrogen)

Validation

All antibodies were validated by their manufactures and further confirmed by preliminary experiments with appropriate control samples. The manufacture's validation data are available on their websites: CL8492AP, https://www.biocompare.com/Product-Reviews/187936-Anti-Mouse-Human-Mac-2-Galectin-3-Antibody-CL8942AP/
A-21094, https://www.thermofisher.com/antibody/product/Goat-anti-Rat-IgG-H-L-Cross-Adsorbed-Secondary-Antibody-Polyclonal/A-21094.

Eukaryotic cell lines

Policy information about cell lines and Sex and Gender in Research

Cell line source(s)

E0771 was obtained from CH3 Biosystems. MC-38 was obtained from Kerafast. HEK293T was obtained from Clontech. KUSA-A1, LLC-PK1 were obtained from JCRB Cell Bank. NIH3T3,AML-12, MS1, HeLa, MCF-7, Jurkat were obtained from ATCC. HMF3S is a gift from Dr. Parmjit Jat and previously described in doi: 10.1073/pnas.98.2.646.

Authentication

Auhentifications were provided by commercial providers (CH3 Biosystems / Kerafast), JCRB Cell Bank and ATCC. We also authenticated the cell lines by morphology check and growth curve analysis.

Mycoplasma contamination

All of the cell lines were tested for mycoplasma contamination.

Commonly misidentified lines
(See ICLAC register)

n/a

Animals and other research organisms

Policy information about studies involving animals; ARRIVE guidelines recommended for reporting animal research, and Sex and Gender in Research

Laboratory animals

Five week-old C57BL/6 Albino female mice were obtained from Charles River Laboratory Japan. All mice used were provided access to food and water ad libitum, and were housed in the animal facilities at Tokyo Institute of Technology, RIKEN or Jichi Medical University. The animal facilities were maintained at 20-25 ºC with 40-60% humidity under a standard 12-hour light-dark cycle.

Wild animals

No wild animals were used in the study.

Reporting on sex

This study specifically used female mice because a high incidence of breast cancer are clinically diagnosed in female patients.

Field-collected samples

No field collected samples were used in the study.

Ethics oversight

Ethical approval of this study protocol for animal experiments was obtained from Tokyo Institute of Technology, Jichi Medical University, and RIKEN. The maximal tumor size permitted by our ethics committees or institutional review boards was 20 mm at the largest diameter in mice and was not exceeded in our experiments.

Note that full information on the approval of the study protocol must also be provided in the manuscript.

Plants

Seed stocks	n/a
Novel plant genotypes	n/a
Authentication	n/a

Flow Cytometry

Plots

Confirm that:

- [x] The axis labels state the marker and fluorochrome used (e.g. CD4-FITC).
- [x] The axis scales are clearly visible. Include numbers along axes only for bottom left plot of group (a 'group' is an analysis of identical markers).
- [x] All plots are contour plots with outliers or pseudocolor plots.
- [x] A numerical value for number of cells or percentage (with statistics) is provided.

Methodology

Sample preparation	The cell lines and dissociated liver cells were maintained on the ice before flow cytometry analysis. These cells were measured after filtering through a 35-µm strainer cap.
Instrument	SH800 (SONY), FACS Aria (Becton, Dickinson and Company)
Software	Cell Sorter Software (SH800), BD FACSDiva8.0.2 (FACS Aria), FlowJo 10.8.1
Cell population abundance	This study adopted appropriate gating strategies to robustly detect distinct cell population as shown in Figs. S6 and S12. For quantitative analysis, 10000 single cells were analyzed at least. The instruments were thoroughly cleaned to ensure no counts detected from debris.
Gating strategy	For in vitro sGRAPHIC labeling, cells were firstly gated on FSC vs SSC and then FSC-H vs FSC-W to extract single cells. The single cells were then gated on mCherry vs GFP to extract the GFP-positive population with or without the mCherry signal. For in vivo sGRAPHIC labeling, cells were firstly gated on FSC vs SSC, FSC-H vs FSC-W and then SSC-H vs SSC-W to extract single cells. The single cells were then evaluated for their viability by gating on the negative population using Fixable Viability Stain 700. The viable population was gated by mCherry vs GFP to sort the mCherry-positive population with or without the GFP signal. The gate for a GFP-positive population was determined to exclude all cells in control healthy livers. In addition, the viable population was gated with mCherry-negative and BFP (Azurite)-positive to sort metastasized cancer cells.

- [x] Tick this box to confirm that a figure exemplifying the gating strategy is provided in the Supplementary Information.
