# Supplementary material for: Secretory GFP reconstitution labeling of neighboring cells interrogates cell–cell interactions in metastatic niches
Source: Nat Commun. 2023 Dec 5;14:8031. doi: 10.1038/s41467-023-43855-2 (PMC10697979; doi:10.1038/s41467-023-43855-2)
Supplement: Supplementary file 4 — Description of Additional Supplementary Files [file 41467_2023_43855_MOESM4_ESM.pdf]

Description of Additional Supplementary Files

File Name: Supplementary Movie 1
Description: Time-lapse fluorescence imaging of co-culturing MC-38/sC-GR and NIH3T3/N-GR. We co-cultured MC-38 and NIH3T3 at a ratio of 1:1.

File Name: Supplementary Movie 2
Description: Time-lapse fluorescence imaging of co-culturing E0771/sC-GR and NIH3T3/N-GR. We co-cultured E0771 and NIH3T3 at a ratio of 1:1.

File Name: Supplementary Movie 3
Description: Time-lapse fluorescence imaging of co-culturing MC-38/sC-GR and AML12/N-GR. We co-cultured MC-38 and AML12 at a ratio of 1:50.
